# Supplementary material for: Repurposing the aldose reductase inhibitor and diabetic neuropathy drug epalrestat for the congenital disorder of glycosylation PMM2-CDG
Source: Dis Model Mech. 2019 Nov 11;12(11):dmm040584. doi: 10.1242/dmm.040584 (PMC6899038; doi:10.1242/dmm.040584)
Supplement: Supplementary information [file dmm-12-040584-s1.pdf]

Supplementary Figure 1

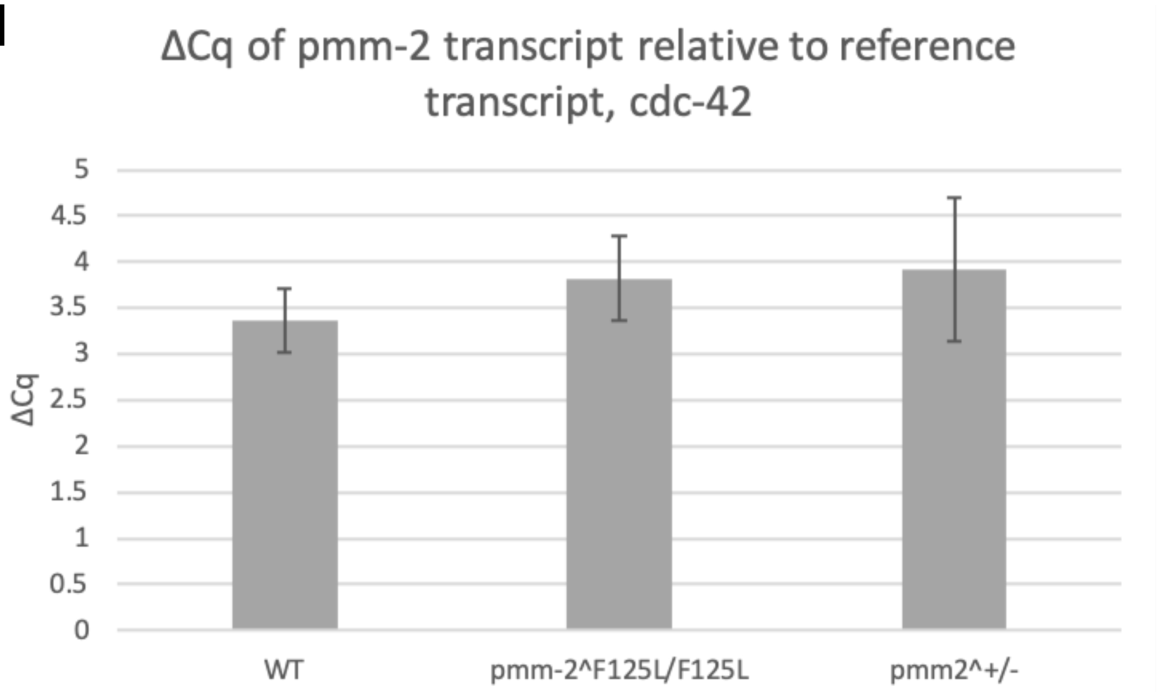

Figure S1. Quantitative RT-PCR analysis of PMM-2 expression in pmm-2 F125L (F119L) homozygote mutant worms compared to the heterozygous *pmm-2* deletion mutant and a wildtype reference strain (N2). ΔCq values were calculated by subtracting the reference transcript (cdc-42) cycle quantification (Cq) number from that of the target transcript (pmm-2). From left to right, ΔCq values for N2, *pmm-2* F125L/F125L homozygote mutant (COP1626) and pmm-2 heterozygous mutant (VC3054) are displayed. Each bar consists of data from three biological replicates. Error bars are standard error of mean ΔCq across three replicates ΔCq values are not significantly different from wildtype indicating that both homozygous and heterozygous mutants produce the same level of *pmm-2* transcript as N2.

Supplementary Figure 2

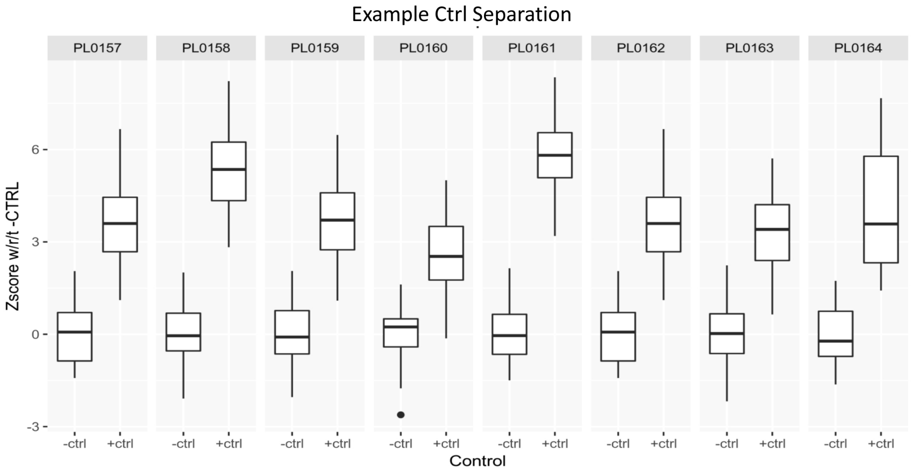

Figure S2. Box and whisker plots of z scores of positive and negative control wells from a representative replicate of the Microsource Spectrum drug repurposing library screen. Negative controls have Z score values of 0, whereas Z score values of positive controls are > 2.

Disease Models & Mechanisms • Supplementary information

Supplementary Figure 3

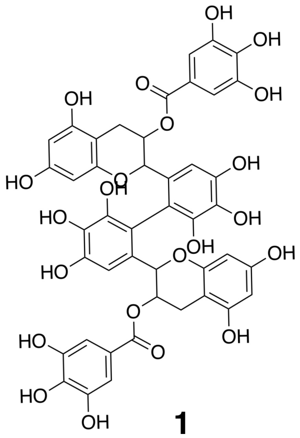

1

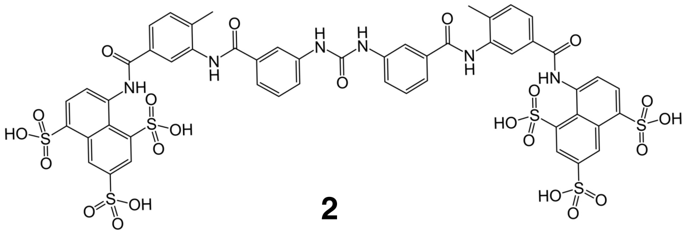

2

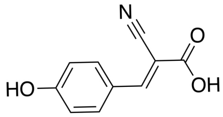

3

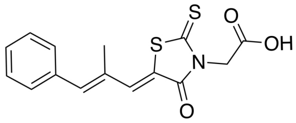

4

Figure S3. Chemical structures of yeast repurposing candidates from Lao et al, 2019 (**1**) 2'-2'-bisepigallocatechin digallate (**2**) suramin (**3**) alpha-cyano-4-hydroxycinnamic acid, and the chemical structure of the aldose reductase inhibitor epalrestat (**4**). Notice the shared carboxylic moiety of **3** and **4**.

Supplementary Figure 4

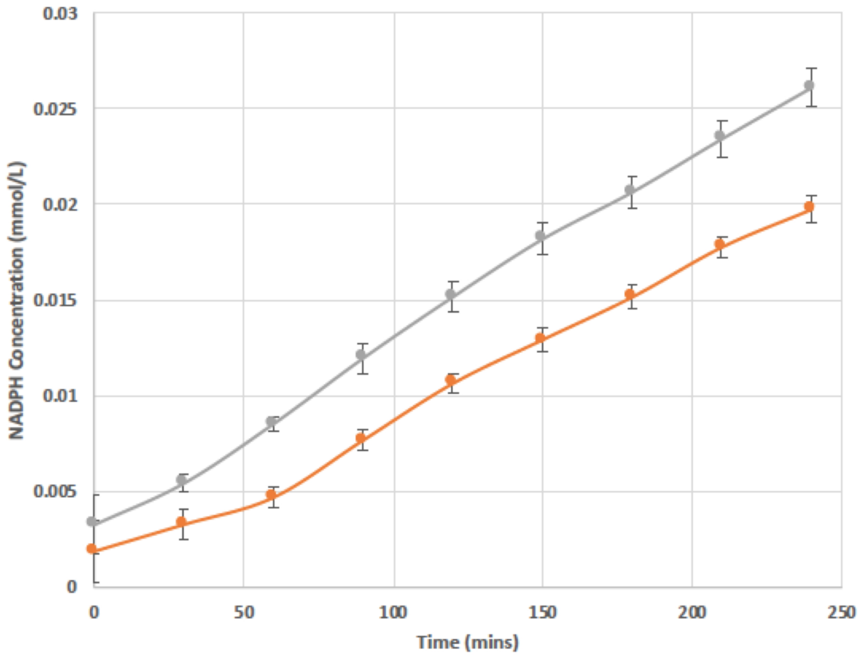

Figure S4. PMM2 enzymatic activity assay of R141/F119L PMM2-CDG patient fibroblasts. Supplemented samples were treated with 10µM epalrestat for 24 hour. Error bars in the bar graphs indicate standard error.

Supplementary Figure 5

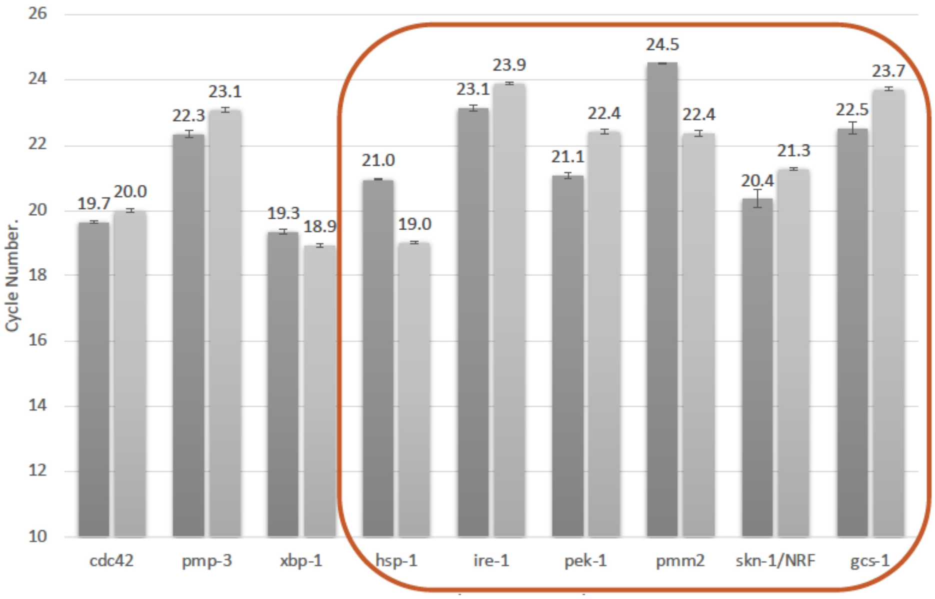

Figure S5. Quantitative RT-PCR analysis of PMM-2 and ER stress marker expression in *pmm-2* F125L/F125L homozygote mutant worms.

Disease Models & Mechanisms • Supplementary information

Supplementary Figure 6

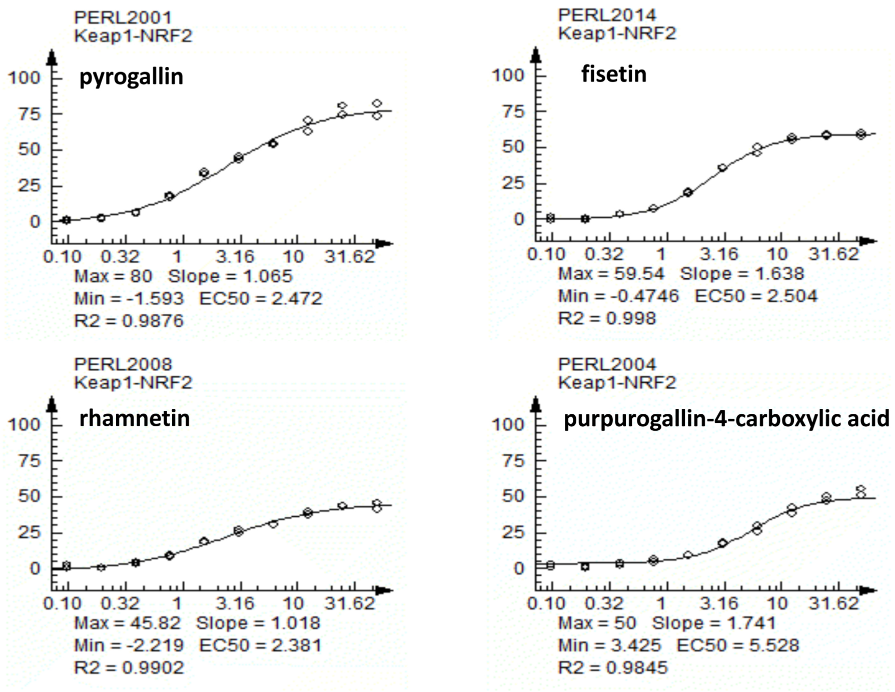

Figure S6. Dose-response curves of four worm PMM2 repurposing hits in a Keap1-NRF2 reporter activation assay in human cells generated by DiscoverX. (**A**) pyrogallin. (**B**) fisetin. (**C**) rhamnetin. **D**) purpurogallin-4-carboxylic acid.

Disease Models & Mechanisms • Supplementary information

Supplementary Figure 7

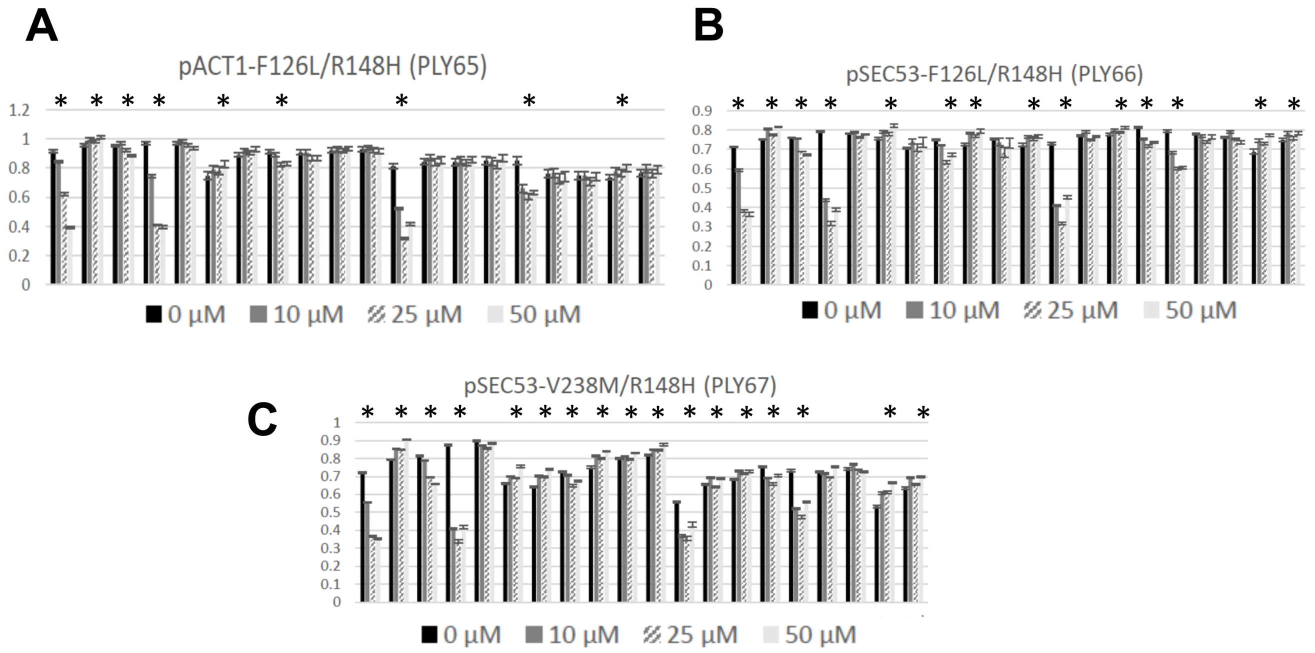

Figure S7. Growth of three yeast PMM2-CDG models (pACT1-F126L/R148H; pSEC53-V238M/R148H; pSEC53-F126L/R148H) in the presence of the 20 worm repurposing candidates at 10µM, 25µM and 50µM. Compounds tested from left to right: pyrogallin, amidol, baicalein, purpurogallin-4-carboxylic acid, gossypetin, quercetin tetramethylether, 3-methoxycatechol, rhamnetin, theaflavin monogallate, hieracin (tricetin), epicatechin monogallate, 3,4-didesmethyl-5-deshydroxy-3-ethoxyschleroin, 2,3,4-trihydroxy-4-methoxybenzophenone, koparin, fisetin, edaravone, ellagic acid, levodopa, dobutamine and ethylnorepinephrine. Asterisks indicate compounds that have either positive or negative effects on growth.

Disease Models & Mechanisms • Supplementary information

Supplementary Figure 8

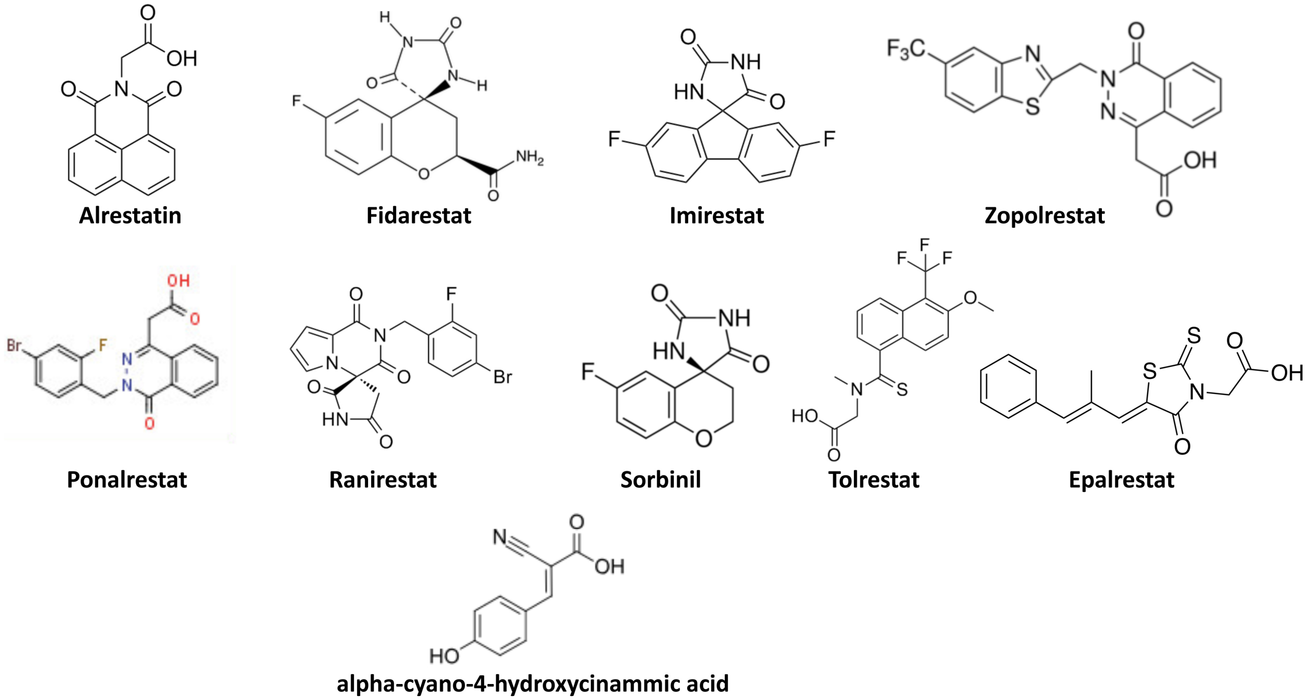

Figure S8. Chemical structures of 10 commercially available aldose reductase inhibitors tested in PMM2-CDG R141H/F119L fibroblasts.

Disease Models & Mechanisms • Supplementary information
